# Gallstone Ileus: A Case Report of an Uncommon Cause of Bowel Obstruction in the Emergency Department

**DOI:** 10.7759/cureus.112038

**Published:** 2026-07-04

**Authors:** Christian G Orama Segarra, Michael Torres Silva, Andrea P Quiles, Miguel F Agrait Gonzalez

**Affiliations:** 1 Emergency Medicine, Centro Médico Episcopal San Lucas, Ponce, PRI; 2 Emergency Medicine, Ponce Health Sciences University, Ponce, PRI; 3 Surgery, Centro Médico Episcopal San Lucas, Ponce, PRI

**Keywords:** ct (computed tomography) imaging, emergency medicine, enterolithotomy, gallstone ileus, small bowel obstruction

## Abstract

Gallstone ileus is an uncommon cause of mechanical small bowel obstruction that most often affects older adults with comorbid disease and may be difficult to diagnose because symptoms and laboratory findings are frequently nonspecific. We report the case of a 70-year-old man with hypertension, hypercholesterolemia, coronary artery disease, and gastroesophageal reflux disease who presented to the emergency department with progressive abdominal pain, distention, and recurrent non-bilious emesis after being discharged from another facility following symptomatic treatment. On examination, he had abdominal distention, decreased bowel sounds, diffuse tenderness, and signs of dehydration. Laboratory testing showed no leukocytosis, but lactate was mildly elevated. Computed tomography of the abdomen and pelvis demonstrated a small bowel obstruction caused by a 3 × 2 cm calcified intraluminal mass impacted at the ileocecal valve, consistent with gallstone ileus. Surgical consultation was obtained, and the patient underwent enterolithotomy, with removal of the obstructing gallstone and bowel decompression. Given his age, cardiovascular comorbidities, and elevated operative risk, cholecystectomy and fistula repair were deferred. His postoperative course was uncomplicated, and follow-up showed no recurrent symptoms, with spontaneous closure of the biliary-enteric fistula. This case underscores that gallstone ileus must remain an important diagnostic consideration in older adults presenting with bowel obstruction, even in the absence of known biliary disease. Early CT imaging is essential for timely diagnosis, and operative management should be tailored to the patient’s physiologic risk and overall surgical candidacy.

## Introduction

Gallstone ileus is an uncommon cause of mechanical bowel obstruction that presents diagnostic and therapeutic challenges in emergency medicine. It typically occurs as a late complication of cholelithiasis, when chronic inflammation and adhesion between the gallbladder and adjacent bowel lead to the formation of a biliary-enteric fistula. This permits migration of a gallstone into the gastrointestinal tract, where it may become impacted and cause obstruction, most commonly at the ileocecal valve [1,2].

Gallstone ileus accounts for approximately 1%-4% of mechanical bowel obstructions in the general population, but its relative contribution increases substantially among older adults, with historical series reporting up to 25% of nonstrangulated small bowel obstructions in patients older than 65 years [2,3]. Presentation is often nonspecific and may include abdominal pain, distention, nausea, vomiting, and dehydration. A prior history of symptomatic biliary disease may be absent, which can contribute to delayed diagnosis as it did in our case [1,2].

Definitive management usually requires surgical intervention to relieve the obstruction, but the optimal operative strategy remains individualized. Options include enterolithotomy alone, one-stage enterolithotomy with cholecystectomy and fistula repair, or a staged approach in selected patients [4]. We present the case of an older adult without known biliary disease who developed gallstone ileus, causing small bowel obstruction after an initially missed presentation, highlighting the emergency department's role of early CT imaging and risk-adapted surgical consultation. Delays in care for mechanical small bowel obstructions increase the risk for complications and morbidity, with increasing potential for bowel ischemia [1,2].

## Case presentation

A 70-year-old man with a medical history of hypertension, hypercholesterolemia, coronary artery disease, and gastroesophageal reflux disease presented to the emergency department with worsening abdominal pain and multiple episodes of non-bilious, non-bloody emesis. His symptoms began one day prior as vague abdominal discomfort and abdominal fullness that eventually progressed to severe abdominal pain and distention. He reported subjective fevers and chills but denied diarrhea, recent travel, sick contacts, or similar prior episodes. Before presenting to our emergency department, he had been evaluated at another facility that same day, treated symptomatically, and discharged home. At that outside evaluation, no lab testing or imaging was performed, and the patient was discharged with a diagnosis of gastritis.

On arrival at our facility, the patient appeared in moderate distress due to significant pain and had active emesis. Physical examination revealed dry oral mucosa, decreased bowel sounds, abdominal distention, and diffuse abdominal tenderness with voluntary guarding, most pronounced in the left lower quadrant. He remained hemodynamically stable and afebrile. Laboratory evaluation demonstrated no leukocytosis, although serum lactate was mildly elevated at 2.72 mmol/L (Table 1).

**Table 1:** Patient's laboratory findings BUN: blood urea nitrogen; AST: aspartate aminotransferase; ALT: alanine transaminase

	Results	Reference values
White Blood Cell	10.69	4.5-11.0 × 10⁹/L
Hemoglobin	15.5	14-18 g/dL
Hematocrit	45.2	36-52%
Platelet	321	150-450 × 10⁹/L
Neutrophil %	84%	45-80%
Lactate	2.72	0.5-2.2 mmol/L
Sodium	139	136-145 mmol/L
Potassium	5.1	3.5-5.0 mEq/L
Chloride	102	98-106 mmol/L
CO2	25.2	22-29 mEq/L
BUN	31	10—20 mg/dL
Creatinine	2.30	0.6-1.3 mg/dL
Glucose	163	70-106 mg/dL
AST	27	8-48 U/L
ALT	19	7-56 U/L
Lipase	31	10-151 U/L
Total Bilirubin	1.17	0.2-1.2 mg/dL

Given the patient's progressive abdominal distention, recurrent emesis, decreased bowel sounds, and diffuse tenderness, mechanical bowel obstruction was suspected. Laboratory findings of mild lactate elevation along with elevated creatinine were thought to be due to dehydration and potentially early bowel ischemia from the suspected obstruction. CT imaging of the abdomen and pelvis was then obtained, which demonstrated dilated small bowel loops with a transition point in the distal ileum/ileocecal region caused by a 3 x 2 cm calcified intraluminal mass, consistent with an ectopic gallstone and gallstone ileus (Figures 1, 2). Pneumobilia and a biliary-enteric fistula were not specifically reported on CT.

**Figure 1:**
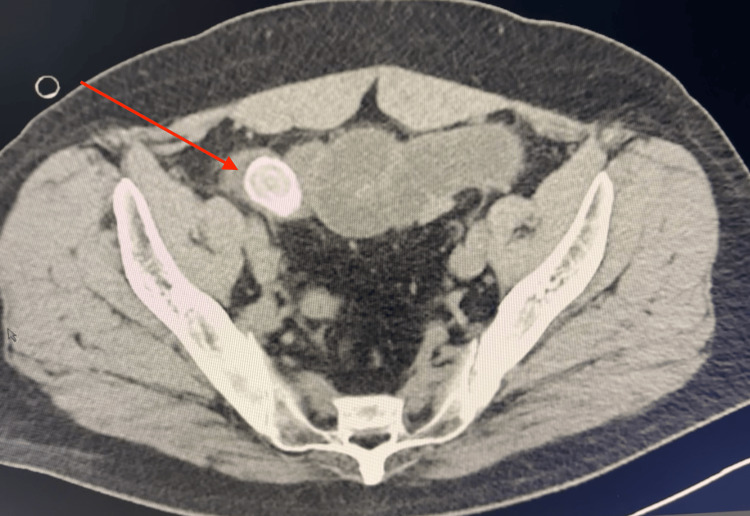
Axial view of the CT scan of the abdomen and pelvis showing a calcified gallstone in the terminal ileum (red arrow) Red arrow: calcified gallstone, CT: computed tomography

**Figure 2:**
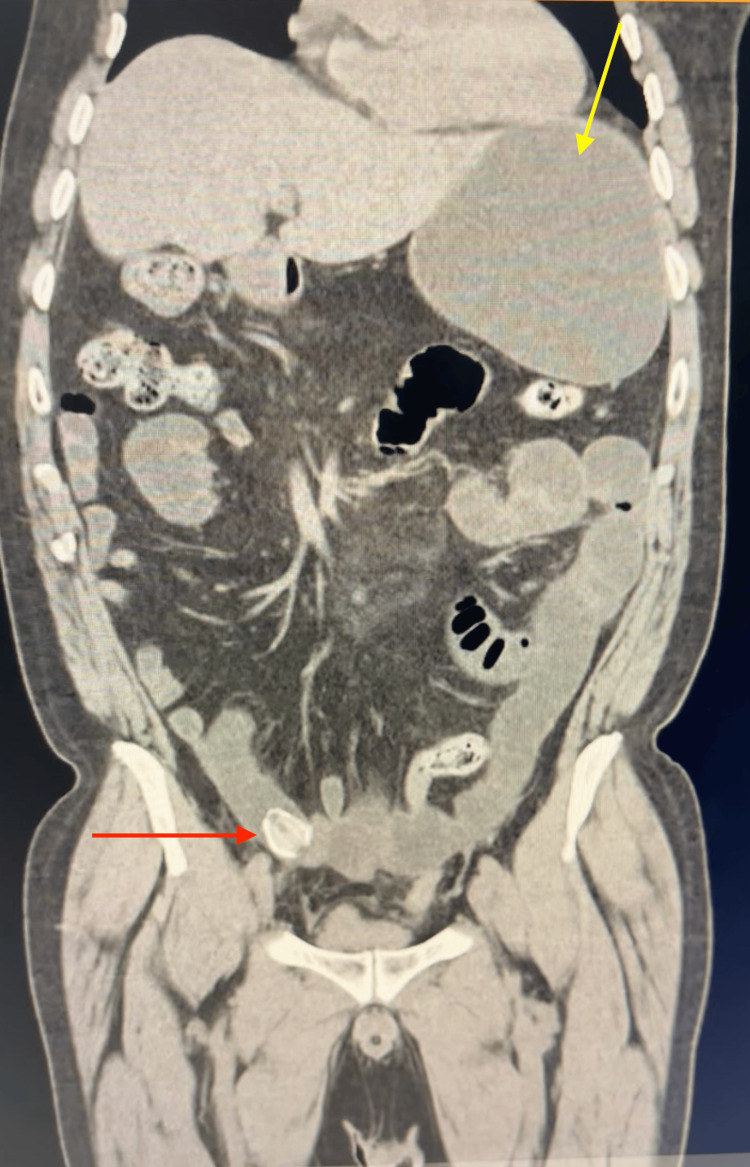
Coronal CT image demonstrating an intraluminal gallstone (red arrow) impacted at the ileocecal valve with stomach and small bowel dilation (yellow arrow) proximal to the stone CT: computed tomography

These findings prompted urgent surgical consultation along with aggressive IV rehydration with lactated Ringer's 30ml/kg bolus and maintenance fluids at 150ml/hr. The surgical service evaluated the patient, and operative intervention was pursued after informed consent was obtained. Because of his American Society of Anesthesiologists (ASA) physical status classification of III-E [5], advanced age, and multiple medical comorbidities, a risk-adapted operative strategy was selected. Enterolithotomy was performed with successful removal of the obstructing gallstone and decompression of the bowel. Intraoperative inspection of the small bowel did not reveal additional obstructing gallstones or any bowel necrosis. Cholecystectomy and biliary-enteric fistula repair were deferred for a later date.

The patient's in-hospital postoperative course was uncomplicated. He demonstrated gradual clinical improvement, tolerated diet advancement, and was discharged home in stable condition, tolerating a normal diet. Surgical follow-up demonstrated no recurrent obstructive symptoms and no need for interval biliary intervention, with the surgical team suspecting a spontaneous fistula closure. The patient was then contacted 12 months post-intervention and stated he has had no further episodes of abdominal distention and has not required a second surgical procedure.

## Discussion

Gallstone ileus is a rare but important cause of small bowel obstruction, particularly among older adults with comorbid disease [1-4]. Although it occurs more commonly in women, it can also present in men and should remain in the differential diagnosis for older patients with bowel obstruction symptoms. The clinical presentation is frequently nonspecific and may mimic more common etiologies of obstruction, including postoperative adhesions, hernia, malignancy, or inflammatory bowel disease. Symptoms may also wax and wane as the stone migrates through the gastrointestinal tract, a pattern sometimes described as the "tumbling" phenomenon [1,4]. In this case, the diagnostic challenge was illustrated by the patient's prior discharge after symptomatic treatment and subsequent presentation with progressive obstructive symptoms.

This case highlights the importance of CT imaging when gallstone ileus or obstruction is suspected, but classic findings are incomplete or absent. Rigler's triad classically consists of bowel obstruction, pneumobilia, and an ectopic gallstone [6]. However, all three findings are not consistently identified, and the absence of reported pneumobilia or a visible fistula should not exclude the diagnosis. CT is the preferred diagnostic modality because it can identify the level of obstruction, characterize the ectopic gallstone, and evaluate for complications such as ischemia or perforation. Reported CT performance for gallstone ileus is high, with a sensitivity of approximately 90%-93%, a specificity of 100%, and an accuracy of 99% in patients presenting with acute small bowel obstruction [7,8]. In this patient, CT demonstrated the clinically decisive findings: small bowel obstruction with a calcified intraluminal gallstone impacted distally.

Surgical management of gallstone ileus remains individualized. The immediate priority is relief of the mechanical obstruction. Operative options include enterolithotomy alone, one-stage enterolithotomy with cholecystectomy and fistula repair, bowel resection when bowel viability is compromised, or delayed biliary surgery in selected patients [4,9,10]. A one-stage operation offers definitive treatment of the gallbladder and fistula, but it may require longer operative time and greater physiologic stress. Enterolithotomy alone is commonly favored in older or higher-risk patients because it addresses the acute obstruction while avoiding the added burden of cholecystectomy and fistula repair during the index operation [9,10].

The operative decision in this case reflected the patient's risk profile. His age, coronary artery disease, ASA III-E classification, dehydration, and elevated lactate favored a focused operation aimed at relieving obstruction and minimizing operative time. The absence of additional stones on intraoperative inspection further supported avoiding more extensive intervention during the acute presentation. This outcome supports a pragmatic emergency surgery approach: early recognition and resuscitation in the emergency department, prompt CT imaging, early surgical consultation, and operative planning tailored to patient risk rather than a uniform surgical strategy.

## Conclusions

Gallstone ileus remains an uncommon but clinically significant cause of mechanical small bowel obstruction in the emergency department, particularly among older adults with comorbid disease and even those with no known history of biliary symptoms. Its nonspecific and sometimes intermittent presentation can lead to diagnostic delay. Early CT imaging is critical when older patients present with abdominal pain, distention, recurrent emesis, decreased bowel sounds, or other features concerning for obstruction. The absence of reported pneumobilia or a visible biliary-enteric fistula should not exclude the diagnosis when CT demonstrates small bowel obstruction with an intraluminal gallstone. Definitive management requires surgical consultation, but the operative approach should be individualized. In high-risk patients, enterolithotomy alone may provide effective obstruction relief while avoiding the added physiologic burden of immediate cholecystectomy and fistula repair.
